# Current Body Mass Index Is Associated with Reported Weight Gain as a Reason for Discontinuing Oral Contraceptive Pill Use

**DOI:** 10.3390/obesities6020020

**Published:** 2026-03-29

**Authors:** Adnin Zaman, Myla Strawderman, Susan W. Groth, Barbara Lohse, Wendy Vitek, Roland J. Thorpe, Elizabeth Heitman

**Affiliations:** 1 Division of Endocrinology, Diabetes and Metabolism, Department of Medicine, University of Rochester Medical Center, Rochester, NY 14642, USA; 2 Division of Endocrinology, Metabolism and Diabetes, Department of Medicine, University of Colorado Anschutz Medical Center, Aurora, CO 80045, USA; 3 Department of Biostatistics and Computational Biology, University of Rochester Medical Center, Rochester, NY 14642, USA; 4 School of Nursing, University of Rochester, Rochester, NY 14642, USA;; 5 Wegmans School of Health and Nutrition, Rochester Institute of Technology, Rochester, NY 14623, USA;; 6 Department of Obstetrics and Gynecology, SUNY Upstate Medical University, Syracuse, NY 13210, USA; 7 Boston IVF—The Syracuse Center, Syracuse, NY 13214, USA; 8 Johns Hopkins Center for Health Disparities Solutions, Johns Hopkins Bloomberg School of Public Health, Baltimore, MD 21205, USA; 9 Program in Ethics in Science and Medicine, University of Texas Southwestern Medical Center, Dallas, TX 75390, USA

**Keywords:** obesity, body mass index, oral contraception, weight gain, patient perceptions

## Abstract

Concerns about weight gain are commonly cited with combined oral contraceptive pill (COCP) use, yet it remains unclear whether perceived weight gain as a reason for discontinuation differs by body mass index (BMI). We analyzed data from the 2017–2019 National Survey of Family Growth (NSFG), including 3709 non-pregnant women aged 20–49 years who had ever used COCPs and had BMI calculated from self-reported height and weight. Trained NSFG staff interviewed participants on reasons for discontinuation and coded them into predefined categories, including weight gain. Discontinuation was examined by BMI category (underweight, normal weight, overweight, obesity) using survey-weighted logistic regression adjusted for demographic and socioeconomic covariates. Overall, 35.2% (95% CI 32.3–38.1%) of women reported discontinuing COCPs due to dissatisfaction, with 20.2% (95% CI 18.1–22.3%) citing side effects. Weight gain was reported by 7.0% (95% CI 5.6–8.4%) of ever-users, with higher prevalence among women with overweight (8.4%) and obesity (7.7%) compared with normal-weight women (5.5%). In adjusted analyses, women with overweight (aOR 1.76, *p* = 0.048) and obesity (aOR 1.68, *p* = 0.033) had higher odds of COCP discontinuation due to self-reported weight gain. These findings highlight the importance of addressing weight-related concerns during contraceptive counseling, particularly for women with higher BMI.

## 1. Introduction

Combined oral contraceptive pills (COCPs) are one of the most commonly used reversible contraceptive methods among reproductive-aged women in the United States (U.S.) [1]. Despite their effectiveness, many women discontinue COCPs prematurely due to perceived or actual side effects, including weight gain [2–5]. Although previous clinical trials have not consistently shown significant weight gain with COCP use [6–9], women’s weight-related concerns continue to influence their contraceptive decision-making and discontinuation [10]. Perceived weight gain may occur even in the absence of objectively measured weight change, potentially influencing contraceptive experiences differently across individuals.

Perceptions of weight gain may be particularly salient for individuals with a higher body mass index (BMI). Specifically, women with overweight or obesity often face societal stigma and may therefore experience heightened concern about body image, making them more sensitive to possible changes in weight [11,12]. Contraceptive uptake behaviors vary by BMI status such that women with obesity are less likely to use systemic hormonal methods and more likely to rely on sterilization or long-acting reversible contraception (LARC) instead of shorter-acting methods like COCPs [13–15]. These patterns of contraceptive initiation have been attributed in part to concerns about weight gain but also to self- or provider-perceived limitations in the safety or efficacy of COCPs among those at higher body weights [15–17]. However, to our knowledge, no studies have directly examined whether the likelihood of discontinuing COCPs due to perceived weight gain differs by BMI.

In this study, we used data from the 2017–2019 National Survey of Family Growth (NSFG)—a sample representing over 70 million U.S. women—to investigate whether perceived weight gain as a reason for COCP discontinuation differs by BMI category. We hypothesized that women with overweight or obesity would be more likely to cite weight gain as a reason for discontinuing COCP use than normal-weight women.

## 2. Materials and Methods

### 2.1. 2017–2019 National Survey of Family Growth Dataset

The NSFG is an ongoing, federally funded, nationally representative survey conducted by the Centers for Disease Control and Prevention’s National Center for Health Statistics (NCHS). It utilizes confidential, voluntary, in-person, computer-assisted personal interviews (CAPIs) administered by trained interviewers [18]. The dataset originated in 1973 and initially collected information periodically but transitioned to a continuous survey design in 2006. Since 2015, the NSFG has collected data from men and women aged 15–49 years about fertility, family planning, and reproductive health—including contraceptive practices. Although the survey operates continuously, data are released in 2-year intervals to provide manageable datasets for public use and to ensure adequate sample sizes for analysis [18].

Respondents to the NSFG were selected through a stratified, multistage probability sample of the U.S. household population, with oversampling of Hispanic/non-Hispanic Black adults and adolescents to improve subgroup estimates. All interviews were conducted in person by trained female interviewers using CAPIs. More sensitive items were administered via audio computer-assist self-interview (CASI). A total of 11,347 interviews were completed for the 2017–2019 dataset, consisting of 6141 women and 5206 men [19].

The present cross-sectional analysis utilized data from the 2017–2019 NSFG dataset. Our analytic sample included non-pregnant women aged 20–49 years who had ever used COCPs and had available BMI data. Pregnant women and adolescents (ages 15–19) were excluded as BMI is less appropriate for describing these groups [20,21].

### 2.2. Questionnaires and Data Management

The NSFG questionnaires are organized into thematic sections, with separate codebooks available for male, female, and pregnant respondents. For this study, we used the 2017–2019 Female Respondent File, which contains 11 sections (A–J and W) [22]. Data on contraceptive use and discontinuation were obtained from Section E (*Pregnancy, Contraception, and Reproductive Health*), which includes items on ever-use of contraceptive methods, discontinuation due to dissatisfaction, and structured follow-up questions on specific reasons for discontinuation. Responses were collected using show cards (e.g., cards 30–32) listing standardized options such as “side effects.” After a respondent selected a general category, open-ended follow-up questions were asked; interviewers then coded these open-ended responses into one of 23 predefined reasons, including “weight gain.” All open-ended responses were coded by trained NSFG staff using standardized procedures as described in the 2017–2019 NSFG user documentation; however, coder training materials and interrater reliability metrics are not publicly available.

Demographic and socioeconomic covariates were drawn from other sections of the Female Respondent File, including Section A (*Household*), Section B (*Respondent Background*), and Section C (*Marital and Relationship History*). Public-use datasets, documentation, and codebooks are available through the NCHS website [23].

### 2.3. Variables

Independent Variable: BMI was computed by the NCHS using NSFG respondents’ self-reported height and weight with the equation: *BMI = (Weight in pounds/Height in inches*^2^*)* × *703*. This continuous BMI variable was rounded to one decimal place and then reported as BMI categories—Underweight (<18.5 kg/m^2^), Normal Weight (≥18.5–24.9 kg/m^2^), Overweight (≥25.0–29.9 kg/m^2^), and Obese (≥30.0 kg/m^2^)—to preserve respondent anonymity during public data use [24]. These categories reflect standard CDC cut points and are consistent with prior NSFG publications [25].

**Figure 1.**
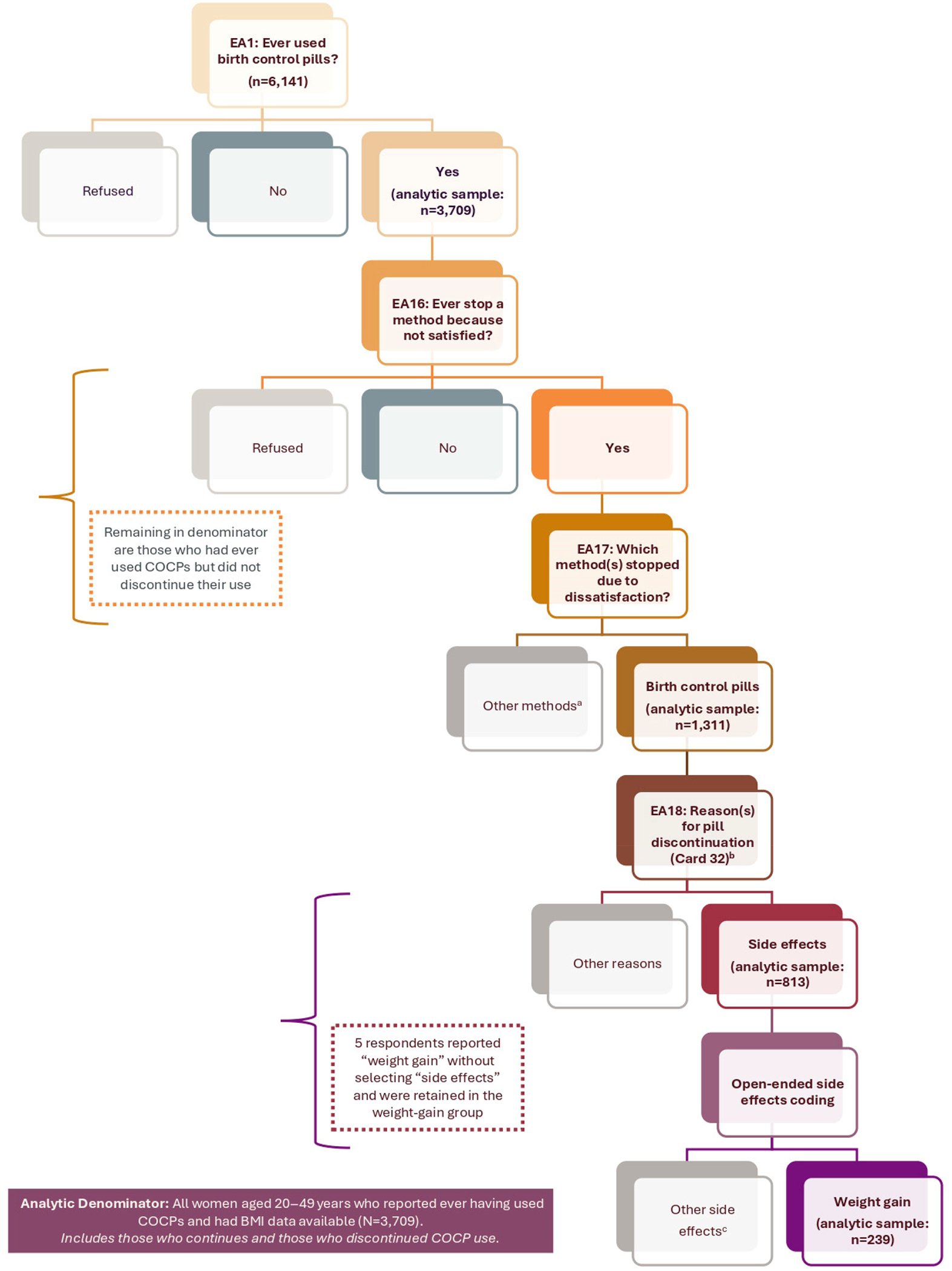
Flow diagram of analytic sample selection and outcome classification in the 2017–2019 NSFG dataset. The analytic sample included non-pregnant women aged 20–49 years with available BMI data who reported ever using COCPs (n = 3709). The NSFG question structure is shown for context; all reported counts correspond to this analytic sample unless otherwise specified. Among these women, 1311 reported discontinuing COCP use for any reason. Of those, 813 reported side effects as a reason for discontinuation, and 239 reported weight gain as a reason based on coded open-ended responses. Five respondents reported weight gain without selecting “side effects” and were retained in the weight gain group. ^a^ EA17 asked, “Please look at Card 31. What method or methods did you stop because you were not satisfied?” Card 31 included the following options: Birth control pills, Calendar rhythm/Standard Days/Cycle Beads Method, Cervical cap, Condom, Contraceptive patch (Ortho-Evra or Xulane), Depo-Provera (shot), Diaphragm, Female condom/vaginal pouch, Female sterilizing operation (such as tubal sterilization or hysterectomy), Foam, Hormonal implant (Norplant, Implanon, or Nexplanon), IUD, Jelly or cream, Lunelle injectable (monthly shot), Partner’s vasectomy, Safe period by temperature or cervical mucus test (Two Day, Billings Ovulation, or Sympto-thermal method), Suppository/insert, Today sponge, Vaginal contraceptive ring, Withdrawal/pulling out, Other method. ^b^ EA18 asked, “Looking at Card 32, What was the reason or reasons you were not satisfied with the Pill?” Choices for participants on Card 32 included the following options: Too expensive, Insurance did not cover it, Too difficult to use, Too messy, Your partner did not like it, You had side effects, You were worried you might have side effects, You worried the method would not work, The method failed/you became pregnant, The method did not protect against disease, Because of other health problems/a doctor told you that you should not use the method again, The method decreased your sexual pleasure, Too difficult to obtain the method, Did not like the changes to your menstrual cycle, Other—specify. ^c^ The universe of respondents who selected “too difficult to use,” “side effects,” or “other” reason for discontinuing the pill were asked to provide open-ended response to reason(s) for discontinuation of pill. These reason(s) were then coded into the following pre-specified categories by trained NSFG staff: Can’t remember to take pill (regularly), Interfered with other medications, Weight gain, Headaches/migraines, Mood swings/depression, Bleeding problems (spotting, too much, irregularly), Hair loss, Nausea/sickness, Skin problems, Hormonal problems/changes, Didn’t like/Didn’t want to use, Concerns about risks (such as breast cancer), Weight loss, Menstrual cramps, Leg cramps, Dizziness, Decreased sex drive, High blood pressure, Blood clots, Fatigue, Other side effects, Other (too few cases, not classifiable elsewhere), Not ascertained.

Dependent Variable: COCP discontinuation due to self-reported weight gain was the primary outcome. In the NSFG, respondents who reported prior COCP use were asked about reasons for discontinuation, and responses were coded into standardized categories. The survey therefore captures whether respondents reported weight gain as a reason for COCP discontinuation rather than objectively measured weight change. This variable was captured from reasons for discontinuation compiled in Section E of the Female Respondent File [22]. Respondents who indicated stopping COCPs because of dissatisfaction were asked to specify reasons, either from show card prompts or via open-ended responses. Trained interviewers recorded and coded open-ended responses into 23 predefined reasons—including “weight gain”—with frequencies logged. Figure 1 provides a flowchart illustrating the survey questions and response pathways utilized for defining COCP discontinuation and reasons for discontinuation. The outcome reflects whether respondents ever reported weight gain as a reason for discontinuing COCP use; women who discontinued for other reasons or who did not discontinue COCPs were included in the reference group. All outcome variables were derived among women who had ever used COCPs with available BMI data (n = 3709), corresponding to the analytic denominator shown in Figure 1.

Covariates: Covariates were selected *a priori* based on prior literature connecting demographic and socioeconomic factors to contraceptive use and BMI [26–28]. These included age (continuous), race/ethnicity (Hispanic, non-Hispanic White, non-Hispanic Black, other), marital status (never married, married, separated/divorced, widowed), parity (nulliparous, 1, 2, or 3+), education (<high school, high school graduate, some college, college graduate, post-college), annual household income (<$25,000, $25,000–$74,999, $75,000–$99,999, ≥$100,000), and smoking status (current, former, never). Race and ethnicity were collected separately in the NSFG but are provided as a single combined recoded variable in the public-use dataset. Covariates were derived from Sections A–C of the Female Respondent File, with some variables (e.g., education, poverty index, smoking) obtained from NCHS-created recodes for disclosure protection [24]. These variables were included to account for potential confounding in the estimation of associations.

### 2.4. Statistical Analysis

This study primarily aimed to estimate the association between BMI categories and the odds of discontinuing COCP use because of concerns about weight gain among adult female NSFG respondents who had ever used COCPs. The outcome of interest was the odds of ever reporting weight gain as a reason for COCP discontinuation, rather than modeling all potential discontinuation pathways. Analyses incorporated the stratified, clustered design and unequal sampling probabilities inherent in the NSFG dataset to yield nationally representative estimates [19,25]. SAS software (version 9.4, SAS Institute, Cary, NC, USA) was utilized, employing Survey procedures to account for the complex survey design and sampling weights. Unadjusted survey-weighted proportions (SURVEYFREQ) or means (SURVEYMEANS) of respondent characteristics are described for the overall population as well as within BMI categories. All survey estimates are reported with 95% confidence intervals (CI). Univariate associations between BMI category and other characteristics were evaluated by the modified Rao-Scott Chi-square test or a *t*-test (SURVEYREG).

Adjusted odds ratios (aORs) were estimated using survey-weighted logistic regression (SURVEYLOGISTIC), accounting for the stratified, clustered, and weighted design of the NSFG and controlling for potential confounders including age, race/ethnicity, marital status, parity, education, income, and smoking status [26–28]. Because the prevalence of discontinuation due to perceived weight gain was approximately 7%, odds ratios provide a valid measure of association without substantial overestimation of the relative risk.

## 3. Results

A total of 3709 non-pregnant women aged 20–49 years who had ever used COCPs and who had available BMI data were included in our analytic sample. When survey weights were applied, this group corresponded to approximately 46.9 million women in the U.S. household population, representing 64.7% of the 72.3 million women represented by the 6141 female respondents in the 2017–2019 NSFG dataset. Women who were currently pregnant, aged 15–19 years, had missing BMI data, or reported never using COCPs were excluded.

### 3.1. Sample Characteristics

The weighted distribution of BMI among the analytic population was 2.6% underweight, 34.3% normal weight, 27.0% overweight, and 36.2% obese (Table 1). Women with overweight or obesity tended to have children, lower household income, and lower education levels compared to their normal-weight peers. Across all BMI categories, most participants (approximately 60%) identified as non-Hispanic White, with smaller proportions identifying as non-Hispanic Black, Hispanic, or other race/ethnicity.

### 3.2. Reasons for COCP Discontinuation

**Figure 2.**
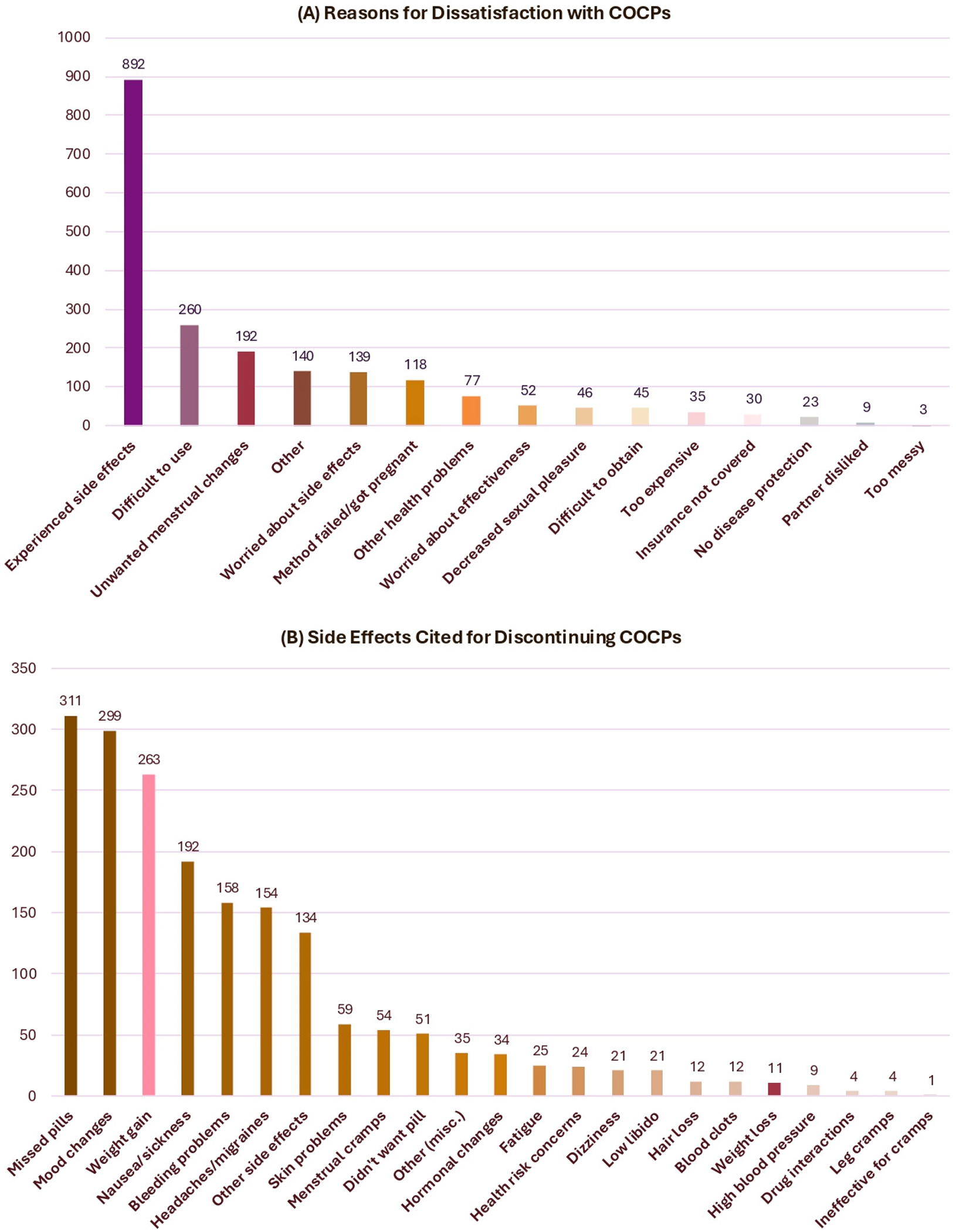
Reasons for dissatisfaction with COCPs (**A**) and specific side effects cited for discontinuation (**B**), NSFG 2017–2019. Reflected in this figure are responses from the broader NSFG respondent universe defined by each survey item and is not restricted to the analytic sample used in regression analyses.

Overall, 35.2% (95% CI: 32.3–38.1%) of eligible women (n = 1311) reported discontinuing COCPs for any reason, corresponding to roughly 16.5 million U.S. women. Among eligible women who discontinued COCPs, 20.2% (95% CI: 18.1–22.3%) cited side effects as the primary reason for discontinuation (Figure 2A). Among all COCP users in our analysis set, 7.0% (95% CI: 5.6–8.4%; n = 239) reported weight gain as a reason for discontinuation, equivalent to approximately 3.3 million U.S. women (Table 2). Respondents could select more than one reason for stopping COCPs, and these reasons were not ranked or ordered by importance. Mood changes or depression were also commonly cited (8.8%) (Figure 2B, Table 2).

Panel A shows responses to EA18 (“Looking at Card 32, what was the reason or reasons you were not satisfied with the pill?”) among 1448 women who reported stopping COCPs due to dissatisfaction for EA17. Participants could select multiple reasons, captured across nine response slots (REASPILL01–REASPILL09).

Panel B shows coded open-ended side effects (STOPPILL1–STOPPILL6) among 1031 women who selected “side effects,” “too difficult to use,” or “other” for EA18 and provided follow-up text responses. Trained NSFG coders classified these responses into 23 predefined categories.

For both panels, simplified labels are displayed for clarity; full response wording is available in the NSFG Female Respondent Codebook E. Because multiple responses were permitted, frequencies represent the number of times each option was selected rather than the number of respondents. Responses were not mutually exclusive.

**Figure 3.**
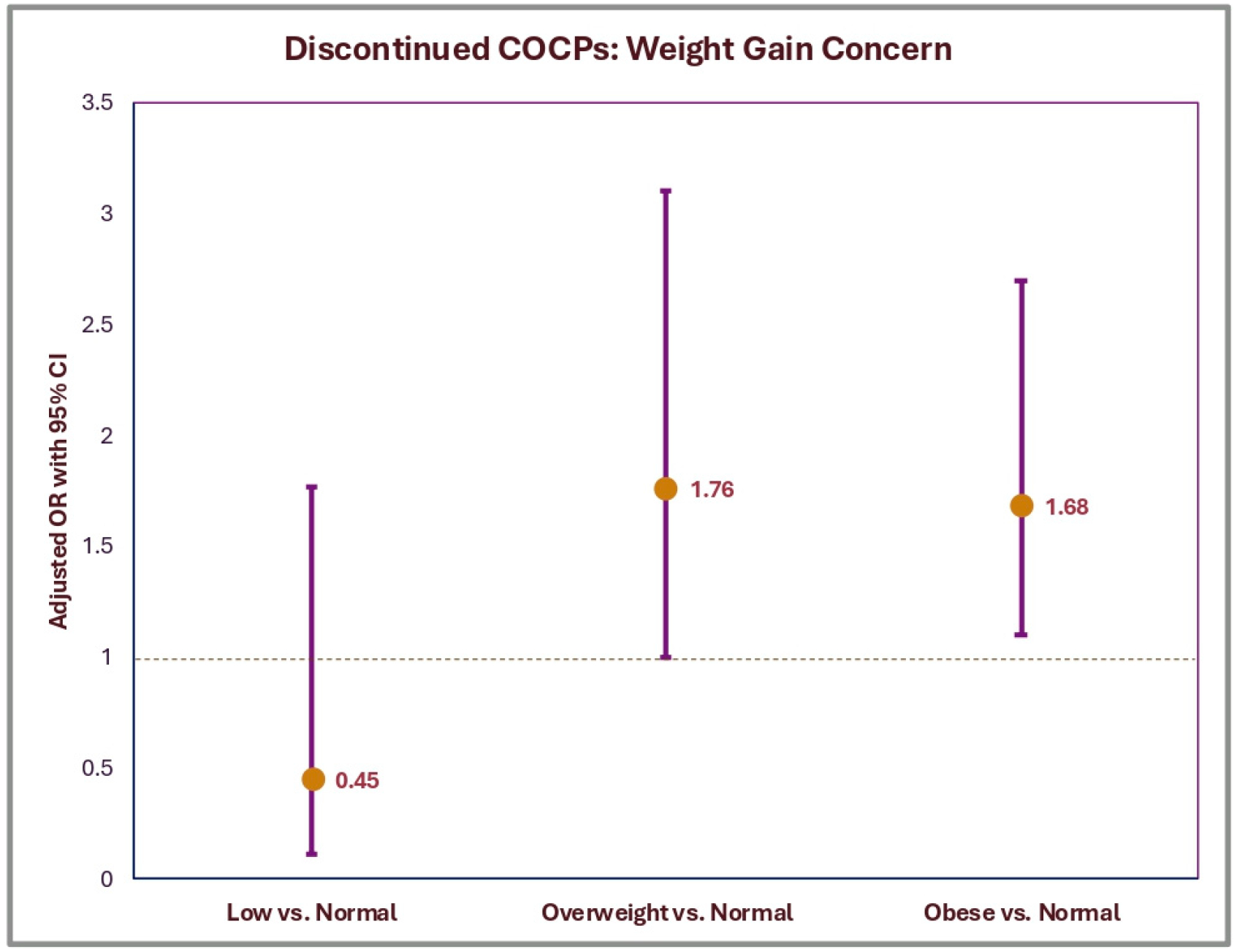
Adjusted odds ratios of discontinuing COCPs due to perceived weight gain by BMI category, NSFG 2017–2019. Logistic regression estimates are shown for the association between BMI category and discontinuation of COCPs due to perceived weight gain. Normal-weight women serve as the reference group. Error bars indicate 95% CIs. Models were adjusted for age, race/ethnicity, parity, marital status, education, income, and smoking status.

When stratified by BMI, weight gain was reported by women in the following categories: 2.6% (95% CI: 0–5.6%) underweight, 5.5% (95% CI: 3.3–7.6%) normal weight, 8.4% (95% CI: 5.3–11.6%) overweight, and 7.7% (95% CI: 5.3–10.1%) obese. After adjusting for demographic and socioeconomic factors, the odds of discontinuing COCPs due to concerns about weight gain were significantly higher for women with overweight (aOR = 1.76, 95% CI: 1.0–3.1, *p* = 0.048) and obesity (aOR = 1.68, 95% CI: 1.1–2.7, *p* = 0.033) compared to normal-weight counterparts (Figure 3). Underweight women did not differ significantly from the normal-weight group (aOR = 0.45, *p* = 0.245). No other covariates were statistically significant predictors of discontinuation in the adjusted model (Table 3).

## 4. Discussion

In this nationally representative survey-based analysis, women with overweight or obesity were significantly more likely than women with normal weight to report discontinuing COCPs due to weight gain (aOR~1.7). Although statistically significant, the observed associations were modest, consistent with the multifactorial nature of contraceptive discontinuation. In contrast, discontinuation rates did not differ between underweight and normal-weight women. Only about 7% of women who had ever used COCPs reported discontinuation due to weight gain, compared with nearly 9% who discontinued because of mood changes or depression. Although discontinuation due to weight gain was reported by a minority of users, these findings suggest that weight-related perceptions may disproportionately influence contraceptive discontinuation among women with higher BMI.

COCPs—a type of combined hormonal contraception—are a highly effective method of birth control, especially if used as intended (i.e., “perfect use”) [29]. In addition to their efficacy to prevent pregnancy, COCPs have many other medical applications, such as to treat menstrual irregularities, acne, or premenstrual disorder (PMS) [30]. Despite these benefits, a prevalent perception among the general public—often erroneously perpetuated by mainstream and social media—is that COCPs may negatively affect the body [31–34]. Weight gain is a major concern among COCP users, and in our analysis, women with higher current BMI had greater odds reporting discontinuation of COCPs due to weight gain.

To suggest that COCPs have no possible adverse effects would be inaccurate. Several studies have demonstrated an association of estrogen and progestin-containing contraceptives with adverse physiologic effects, such as increased risk of venous thromboembolism [35–37]. In contrast, the evidence linking COCP use to mood disorders—which was cited as a reason for discontinuation by 8.8% of women in our study—remains mixed [38–40]. The relationship between COCPs and weight changes is even less certain. A 2014 Cochrane review by Gallo et al. [7] found no substantial effect of COCPs on body weight. However, the 49 included studies were of poor quality, compared a variety of COCP formulations, and largely enrolled normal-weight women, thereby limiting their applicability to women with overweight or obesity.

Despite the lack of evidence in the literature linking weight gain with COCP use, concerns about weight gain has been persistent among users. COCPs became available in the U.S. in 1960, and the earliest published report of weight gain as a commonly perceived side effect appeared in 1985, when it was listed among the top three concerns of users [3,30]. A 2018 web-based survey of more than 3000 U.S. women aged 16–50 years, weighted to national demographics, found that 25–33% of COCP users reported weight gain as a side effect [10].

Prior studies have demonstrated that contraceptive use and continuation may differ across BMI groups, with women with overweight or obesity less likely to use COCPs and more likely to rely on sterilization or LARC methods compared to women with normal weight [15,17]. Additionally, weight perception itself may influence choice of contraceptive method, with women in higher BMI categories more likely to select LARC at initiation and more likely to discontinue short-acting hormonal methods, like COCPs [13,14]. Our findings extend this literature by showing that ever reporting COCP discontinuation due to weight gain was more common in those with overweight and obesity.

The observed association between higher BMI and discontinuation because of reported weight gain may reflect a combination of physiologic experiences as well as the psychosocial context of living in a larger body. Women with overweight or obesity often report heightened sensitivity to weight-related changes and may face weight stigma in health care settings [2,11,12]. Perceptions or concerns of weight gain—even when objective changes are absent—may therefore carry greater importance in contraceptive decision-making. In addition, clinicians may hesitate to recommend COCPs for women with obesity because of lingering concerns about reduced efficacy or thrombotic risk [41–43]. Additionally, demographic factors such as age, parity, and reproductive history may influence perceptions of weight-related side effects and contraceptive decision-making. Future studies could explore whether these factors modify the relationship between BMI and reported weight-related discontinuation. Such counseling—if not carefully framed—can unintentionally reinforce patients’ fears that weight gain will make their contraceptive less effective or less safe. Together, these factors may amplify weight-related discontinuation among women with higher BMI.

Our study adds to this body of evidence by showing that women with overweight or obesity were more likely to report having discontinued COCPs due to weight gain than their normal-weight peers. These findings must also be interpreted in the context of clinical guidance. As Morse and Pathak [44] emphasize, contraceptive use remains substantially safer than pregnancy for women with obesity, despite modest pharmacokinetic concerns about COCP effectiveness. Their review underscores the importance of counseling that balances discussion of risks such as venous thromboembolism with reassurance that COCPs remain an acceptable option. More recently, Hammad et al. [45] highlighted best-practice recommendations that weight-related concerns must be explicitly addressed in contraceptive counseling for women with overweight or obesity. Consistent with these recommendations, our findings highlight the importance of addressing weight-related concerns during contraceptive counseling for women with higher BMI. Incorporating anticipatory guidance and shared decision-making into contraceptive counseling may help address concerns about weight or mood-related side effects, fostering informed and sustained COCP use when appropriate.

This study should be interpreted in the context of several limitations. All data in the NSFG were self-reported, which may introduce recall bias or social desirability bias. Because respondents may have discontinued COCPs years prior to survey participation, recall bias may influence reporting of reasons for discontinuation. Participants may not have accurately remembered or may have reinterpreted their reasons for discontinuation over time. This limitation may result in both over- and under-reporting of weight gain as a reason for discontinuation.

Next, BMI was calculated from self-reported height and weight rather than measured anthropometrics, and misclassification across BMI categories is possible. In addition, BMI was measured at the time of the survey and may not reflect BMI at the time COCP use was discontinued. Because BMI can change over time, the observed association represents the relationship between current BMI and retrospective reporting of weight gain as a reason for discontinuation rather than BMI at the time COCP discontinuation occurred. While BMI is widely used, this calculation based solely on height and weight does not capture body composition or fat distribution.

The cross-sectional design precludes causal inference, limiting our ability to determine whether weight perceptions preceded discontinuation or were influenced by other factors. In addition, “weight gain” was only captured if reported by respondents and subsequently coded by NSFG staff, which may underestimate its true prevalence. The NSFG captures respondents’ self-reported reasons for discontinuing COCPs and does not include objectively measured weight change. Additionally, potentially relevant factors such as COCP formulation, duration of use linked to the discontinuation event, prior weight fluctuation, and mental health conditions were not available for inclusion in the analysis and may represent sources of residual confounding. Finally, the NSFG did not include measured weight change with COCP use or information on contraceptive switching behaviors, which limits evaluation of how perceptions compared with objective changes or subsequent method choice.

Despite these limitations, the study has several important strengths and implications for both clinical practice and public health. Although analyses were conducted among 3709 women who had ever used COCPs and had available BMI data, application of NSFG survey weights permits inference to the broader U.S. household population of reproductive-aged women, estimated at approximately 72.9 million. The analysis also focuses on patient-centered reasons for discontinuation, providing real-world insights beyond those typically captured in clinical trials. Importantly, our findings underscore the significance of addressing weight-related concerns during contraceptive counseling.

Clinicians prescribing COCPs to women with overweight or obesity should directly discuss potential concerns about weight gain, clarify existing evidence, and tailor counseling to patient needs. Such approaches are critical to supporting informed contraceptive choice, promoting continued use of effective methods, and reducing unintended pregnancy in this population.

## 5. Conclusions

Self-reported weight gain was a common reason for discontinuation of COCPs, particularly among women with overweight or obesity. Yet, COCPs remain highly effective and accessible contraceptive options and cannot be dismissed solely based on weight concerns in the absence of causal evidence. Importantly, existing studies have not demonstrated a consistent causal link between COCP use and weight gain, underscoring the need for rigorous prospective research to clarify this relationship. In an era where social media often amplify misconceptions about hormonal contraception, clinicians must both counter misinformation with evidence-based counseling and validate patients’ lived experiences. Future research should aim to bridge this gap by conducting prospective studies that measure both actual and perceived weight changes with COCP use, as well as qualitative work to capture women’s perspectives on weight-related side effects. Such studies are especially needed given that women with overweight or obesity have historically been underrepresented or excluded from studies assessing the relationship between weight changes with COCP use. Finally, intervention studies testing patient-centered counseling and education approaches could help mitigate discontinuation driven by weight-related concerns and support sustained contraceptive use.

## Figures and Tables

**Table 1.** Baseline characteristics of women aged 20–49 years in the analytic sample, stratified by BMI category, from the 2017–2019 NSFG dataset. Table headers show the number of respondents (n) and the weighted population size (N) within each column. Weighted means and 95% CI are shown for continuous variables; weighted proportions and 95% CIs are shown for categorical variables. Characteristics include demographic and socioeconomic covariates used in adjusted logistic regression models: age, race/ethnicity, marital status, parity, education, household income, and smoking status. BMI was calculated from self-reported height and weight and categorized according to CDC cut points: underweight (<18.5 kg/m^2^), normal weight (≥18.5–24.9 kg/m^2^), overweight (≥25.0–29.9 kg/m^2^), and obese (≥30.0 kg/m^2^). The weighted BMI distribution was: 2.6% underweight, 34.3% normal weight, 27.0% overweight, and 36.2% obese. Percentages are compared between BMI categories using the modified Rao-Scott Chi-square test while mean age is compared with a *t*-test accounting for the complex survey design.

Characteristic	Overall n = 3709 N = 46,990,082	Underweight n = 94 N = 1,201,800	Normal n = 1174 N = 16,113,033	Overweight n = 974 N = 12,671,796	Obese n = 1468 N = 17,003,452	*p*-Value
**Age (years)**	35.4 (34.9, 35.9)	34.0 (31.2, 36.8)	33.9 (33.0, 34.8)	35.4 (34.8, 36.1)	36.9 (36.2, 37.5)	<0.0001
**Race/Ethnicity**						
*Hispanic*	16.0 (12.3, 19.8)	8.7 (2.0, 15.5)	13.4 (10.1, 16.8)	16.0 (11.5, 20.6)	19.1 (13.3, 24.8)	<0.0001
*Non-Hispanic White*	62.2 (58.1, 66.4)	57.9 (45.4, 70.5)	68.4 (63.7, 73.1)	62.4 (56.8, 68.0)	56.6 (50.8, 62.4)	
*Non-Hispanic Black*	12.5 (9.6, 15.3)	8.5 (0, 18.6)	5.5 (3.9, 7.1)	14.2 (10.2, 18.2)	18.1 (13.5, 22.8)	
*Other*^a^	9.2 (7.2, 11.3)	24.9 (15.1, 34.6)	12.7 (8.7, 16.7)	7.4 (5.1, 9.6)	6.3 (4.6, 7.9)	
**Parity**						
*0 (nulliparous)*	34.4 (31.2, 37.6)	54.9 (40.4, 69.4)	43.2 (38.5, 47.9)	33.2 (28.7, 37.6)	25.6 (21.7, 29.4)	<0.0001
*1*	17.6 (15.8, 19.4)	20.1 (7.8, 32.3)	16.0 (12.8, 19.2)	19.2 (14.7, 23.8)	17.8 (15.0, 20.5)	
*2*	26.2 (24.0, 28.3)	18.2 (7.1, 29.4)	23.0 (18.9, 27.1)	24.5 (20.0, 29.0)	30.9 (27.3, 34.5)	
*3+*	21.8 (19.2, 24.4)	6.8 (1.7, 11.9)	17.8 (14.5, 21.1)	23.1 (19.6, 26.7)	25.7 (21.2, 30.2)	
**Marital Status**						
*Never Married*	37.3 (34.4, 40.3)	52.4 (38.0, 66.7)	40.1 (35.6, 44.6)	35.7 (31.6, 39.7)	34.9 (31.0, 38.8)	0.070
*Married*	47.6 (44.7, 50.4)	33.9 (18.1, 49.6)	46.6 (41.6, 51.7)	50.4 (46.2, 54.5)	47.3 (42.9, 51.7)	
*Separated/Divorced*	14.1 (12.3, 15.9)	13.6 (4.5, 22.6)	12.1 (9.0, 15.2)	13.6 (10.9, 16.4)	16.4 (13.2, 19.5)	
*Widowed*	1.0 (0.5, 1.6)	0.2 (0.0, 0.6)	1.2 (0.1, 2.3)	0.3 (0.0, 0.7)	1.4 (0.3, 2.6)	
**Education**						
<*High School*	5.8 (4.2, 7.4)	2.5 (0, 5.2)	3.6 (2.3, 4.8)	4.3 (2.6, 6.0)	9.3 (6.0, 12.5)	<0.0001
*High School Graduate*	22.8 (20.4, 25.2)	33.0 (18.3, 47.7)	14.9 (11.6, 18.2)	24.5 (20.6, 28.4)	28.3 (23.9, 32.6)	
*Some College*	32.7 (30.4, 34.9)	33.0 (20.5, 45.4)	31.2 (27.2, 35.3)	33.7 (29.2, 38.2)	33.3 (30.2, 36.4)	
*College Graduate*	25.6 (23.2, 28.0)	19.1 (7.3, 30.9)	35.0 (31.9, 38.2)	23.7 (19.3, 28.1)	18.6 (14.8, 22.4)	
*Post-College*	13.1 (11.1, 15.2)	12.5 (3.7, 21.2)	15.3 (12.3, 18.3)	13.8 (10.6, 17.0)	10.6 (7.8, 13.4)	
**Household**						
**Income Level**						
<*$25,000*	21.3 (18.9, 23.7)	34.0 (19.2, 48.8)	16.1 (3.0, 19.1)	20.2 (16.4, 24.0)	26.1 (22.4, 29.9)	<0.0001
≥*$25,000 to $74,999*	42.5 (39.9, 45.1)	42.8 (29.5, 56.1)	39.5 (34.8, 44.2)	42.7 (37.7, 47.7)	45.1 (40.5, 49.8)	
≥*$75,000-$99,999*	12.1 (10.5, 13.7)	8.9 (1.2, 16.6)	15.0 (12.1, 18.0)	11.0 (7.8, 14.2)	10.4 (8.3, 12.6)	
≥*$100,000*	24.1 (20.8, 27.4)	14.3 (4.2, 24.4)	29.4 (23.8, 35.0)	26.0 (21.6, 30.4)	18.3 (14.7, 21.9)	
**Smoking**						
*Current*	21.4 (18.8, 23.9)	32.0 (18.9, 45.2)	20.0 (16.4, 23.6)	22.0 (17.6, 26.3)	21.5 (17.8, 25.1)	0.032
*Former*	12.7 (10.7, 14.7)	1.1 (0, 2.8)	11.2 (8.6, 13.9)	13.9 (11.0, 16.8)	14.1 (11.0, 17.1)	
*Never*	65.8 (62.6, 69.0)	66.8 (53.6, 80.1)	68.6 (64.2, 73.0)	64.0 (59.1, 68.8)	64.4 (60.2, 68.6)	

a Includes Asian, American Indian/Alaska Native, Native Hawaiian/Pacific Islander, multiracial, and other small racial groups as defined by the NSFG recode.

**Table 2.** Prevalence of reasons for COCP discontinuation overall and by BMI category, NSFG 2017–2019. Table shows the number of respondents (n) and the weighted population size (N) within each column. Weighted proportions with 95% CI are shown. “Any dissatisfaction” reflects women who reported stopping COCPs due to dissatisfaction (EA-17). “Side effects” indicates women who cited side effects as their primary reason for discontinuation (EA-18). Specific reasons such as “weight gain” and “mood changes/depression” were coded from open-ended responses to follow-up questions (STOPPILL1–STOPPILL6).

Reason for Discontinuation	Overall % (95% CI) n = 3709 N = 46,990,082	Underweight % (95% CI) n = 94 N = 1,201,800	Normal Weight % (95% CI) n = 1174 N = 16,113,033	Overweight % (95% CI) n = 973 N = 12,671,796	Obese % (95% CI) n = 1468 N = 17,003,452
Any dissatisfaction	35.2	31.6	38.2	35.6	32.3
	(32.3–38.1)	(15.4–47.9)	(34.4–42.0)	(30.9–40.3)	(28.2–36.4)
	n = 1311	n = 29	n = 453	n = 341	n = 488
	N = 16,536,499	N = 379,936	N = 6,155,584	N = 4,509,215	N = 5,491,764
Side effects (primary reason)	20.2	11.7	22.2	21.4	18.0
	(18.1–22.3)	(4.6–18.8)	(18.5–25.9)	(17.4–25.4)	(14.71–21.23)
	n = 732	n = 13	n = 245	n = 209	n = 265
	N = 9,492,621	N = 140,922	N = 3,580,651	N = 2,716,604	N = 3,054,443
Weight gain (coded side effect)	7.0	2.6	5.5	8.4	7.7
	(5.6–8.4)	(0–5.6)	(3.3–7.6)	(5.3–11.6)	(5.3–10.1)
	n = 239	n = 3	n = 55	n = 75	n = 106
	N = 3,289,304	N = 30,005	N = 877,387	N = 1,068,105	N = 1,313,806
Mood changes/depression	8.8	15.2	10.9	8.1	6.9
	(6.9–10.7)	(0–33.5)	(8.2–13.6)	(5.5–10.8)	(4.5–9.3)
	n = 277	n = 10	n = 102	n = 69	n = 96
	N = 4,136,681	N = 182,326	N = 1,756,146	N = 1,031,385	N = 1,166,824

**Table 3.** Survey-weighted multivariable logistic regression of discontinuing COCPs due to perceived weight gain among U.S. women aged 20–49 years, NSFG 2017–2019 (N = 3709). Models account for stratification, clustering, and sampling weights (Taylor-series variance; domain = ever-COCP users with BMI data).

Covariate	aOR	95% CI	*p*-Value
**BMI (kg/m** ^**2**^ **)**			
*Underweight (*<*18.5)*	0.45	0.11–1.77	0.245
*Normal (18.5–24.9)*	ref	ref	ref
*Overweight (25.0–29.9)*	1.76	1.01–3.08	0.048
*Obese (*≥*30.0)*	1.68	1.04–2.72	0.033
**Race/Ethnicity**			
*Hispanic*	1.35	0.77–2.38	0.292
*Non-Hispanic White*	ref	ref	ref
*Non-Hispanic Black*	0.83	0.41–1.69	0.609
*Other*	1.58	0.76–3.30	0.219
**Age (per year)**	0.98	0.95–1.01	0.178
**Parity**			
*0 (nulliparous)*	ref	ref	ref
*1*	1.08	0.56–2.07	0.813
*2*	1.10	0.53–2.26	0.801
*3+*	0.81	0.44–1.50	0.498
**Marital Status**			
*Never Married*	1.08	0.70–1.66	0.720
*Married*	ref	ref	ref
*Separated/Divorced*	1.26	0.70–2.28	0.432
*Widowed*	0.68	0.08–5.45	0.708
**Education**			
<*High School*	0.70	0.27–1.81	0.457
*High School Graduate*	ref	ref	ref
*Some College*	1.34	0.84–2.15	0.215
*College Graduate*	1.28	0.69–2.35	0.424
*Post-College*	1.19	0.47–3.00	0.710
**Household Income**			
<*$25,000*	0.88	0.35–2.21	0.776
*$25,000-$74,999*	0.95	0.46–1.94	0.879
*$75,000–99,999*	ref	ref	ref
≥*$100,000*	0.82	0.40–1.69	0.582
**Smoking (past 12 months)** ^a^			
*Current*	0.88	0.52–1.47	0.608
*Former*	1.27	0.64–2.52	0.491
*Never*	ref	ref	ref

a Smoking status—current, former, or never—is derived from two NSFG survey variables: SMK100 (In your entire life have you smoked at least 100 cigarettes?) and, if yes, SMOKE12 (In last 12 months, how many cigarettes have you smoked/day on average).

## Data Availability

The data analyzed in this study are publicly available from the National Center for Health Statistics (NCHS) National Survey of Family Growth (NSFG) 2017–2019 public-use data files. Documentation and datasets can be accessed at https://www.cdc.gov/nchs/nsfg/nsfg_2017_2019_puf.htm. No new data were generated for this study.

## Abbreviations

The following abbreviations are used in this manuscript:

aOR: Adjusted odds ratio
BMI: Body mass index
CAPI: Computer-assisted personal interview
CASI: Computer-assisted self-interview
CDC: U.S. Centers for Disease Control and Prevention
CI: Confidence interval
COCP: Combined oral contraceptive pill
NCHS: National Center for Health Statistics
NSFG: National Survey of Family Growth
